# Radiomic Detection of Malignancy within Thyroid Nodules Using Ultrasonography—A Systematic Review and Meta-Analysis

**DOI:** 10.3390/diagnostics12040794

**Published:** 2022-03-24

**Authors:** Eoin F. Cleere, Matthew G. Davey, Shane O’Neill, Mel Corbett, John P O’Donnell, Sean Hacking, Ivan J. Keogh, Aoife J. Lowery, Michael J. Kerin

**Affiliations:** 1 The Lambe Institute for Translational Research, National University of Ireland, H91 YR71 Galway, Ireland; m.davey7@nuigalway.ie (M.G.D.); aoife.lowery@nuigalway.ie (A.J.L.); michael.kerin@nuigalway.ie (M.J.K.); 2 Department of Otolaryngology, Galway University Hospitals, H91 YR71 Galway, Ireland; melcorbett@rcsi.ie (M.C.); ivan.keogh@nuigalway.ie (I.J.K.); 3 Department of Breast and Endocrine Surgery, Galway University Hospitals, H91 YR71 Galway, Ireland; shanenoneill@rcsi.ie; 4 Department of Radiology, Galway University Hospitals, H91 YR71 Galway, Ireland; jpglenstal@gmail.com; 5 Department of Pathology and Laboratory Medicine, Warren Alpert Medical School of Brown University, Providence, RI 02903, USA; shacking@cloudpathdiagnostics.com

**Keywords:** thyroid nodules, radiomics, radiogenomics, ultrasound, personalized medicine

## Abstract

Background: Despite investigation, 95% of thyroid nodules are ultimately benign. Radiomics is a field that uses radiological features to inform individualized patient care. We aimed to evaluate the diagnostic utility of radiomics in classifying undetermined thyroid nodules into benign and malignant using ultrasonography (US). Methods: A diagnostic test accuracy systematic review and meta-analysis was performed in accordance with PRISMA guidelines. Sensitivity, specificity, and area under curve (AUC) delineating benign and malignant lesions were recorded. Results: Seventy-five studies including 26,373 patients and 46,175 thyroid nodules met inclusion criteria. Males accounted for 24.6% of patients, while 75.4% of patients were female. Radiomics provided a pooled sensitivity of 0.87 (95% CI: 0.86–0.87) and a pooled specificity of 0.84 (95% CI: 0.84–0.85) for characterizing benign and malignant lesions. Using convolutional neural network (CNN) methods, pooled sensitivity was 0.85 (95% CI: 0.84–0.86) and pooled specificity was 0.82 (95% CI: 0.82–0.83); significantly lower than studies using non-CNN: sensitivity 0.90 (95% CI: 0.89–0.90) and specificity 0.88 (95% CI: 0.87–0.89) (*p* < 0.05). The diagnostic ability of radiologists and radiomics were comparable for both sensitivity (OR 0.98) and specificity (OR 0.95). Conclusions: Radiomic analysis using US provides a reproducible, reliable evaluation of undetermined thyroid nodules when compared to current best practice.

## 1. Introduction

Thyroid nodules occur commonly within the general population, with studies suggesting a prevalence of 20–67%, with an increased propensity in females and the elderly [1,2]. Increased access to healthcare and availability of modern imaging techniques such as ultrasonography (US) have led to the markedly increased detection of thyroid nodules [3]. The American Thyroid Association (ATA), British Thyroid Association (BTA), and European Society for Medical Oncology (ESMO) guidelines recommend US as the primary imaging modality for the assessment of thyroid nodules [4,5,6]. Several classification systems (e.g., ATA, BTA, and Thyroid Imaging Reporting and Data System (TIRADS)) are utilized by radiologists to stratify the risk of malignancy for each thyroid nodule based on US features [4,5,7]. These systems classify lesions on a scale ranging from benign to malignant based on sonographic parameters such as size, echogenicity, degree of margin irregularity, nodule height to width ratio, extra nodular extension, and calcification [4,5,7]. Suspicious nodules then proceed to fine-needle aspiration cytology (FNAC), where they are reassessed and graded as non-diagnostic, benign, atypical, suspicious for malignancy, or definitively malignant, using cytological reporting systems such as the Bethesda or Thy classification systems [8,9]. At present, patients with undetermined nodules following FNAC undergo surgery in order to obtain a definitive histological diagnosis, with 95% of nodules subsequently being stratified as “benign” [10], leading to the conceptualization that the present paradigm is guilty of overexposing patients to unnecessary over-investigation and overtreatment. Thus, it is vital for translational research efforts to focus on means of accurately screening and sub stratifying detected thyroid nodules into benign and malignant categories [11].

Precision medicine builds on the mantra that every patient, cancer, and disease process possesses its own characteristics with individualized diagnoses, prognoses, and management strategies. The clinical application of artificial intelligence (AI) has advanced the field of precision medicine through the exploration of hypotheses in large data sets [12]. Radiomics (or radiogenomics) is a rapidly evolving field that uses AI to extract vast quantities of data from medical imaging [13]. It represents a quantitative approach to medical imaging through mathematical extraction of the spatial distribution of signal intensities and pixel interrelationships, quantifying textural information by using AI analysis methods. Various radiomic methods exist at present, including radiomic AI, machine learning (ML), convolutional neural networks (CNN), and other deep-learning techniques. The radiomic process involves numerous steps incorporating image acquisition, image segmentation, quantitative feature extraction, computational analysis, and finally, computational modeling [14]. Through this use of vast amounts of data, radiomics provides a quick, reproducible, and objective analysis that can inform individualized diagnostics, sub stratification, prognostication, and future management of disease [13,14].

Due to the ever-increasing number of thyroid nodules detected, there is significant interest within the literature to develop novel strategies to inform diagnostics within the clinical workup of thyroid nodules [15]. Current data suggests radiomic imaging analysis may be capable of accurately stratifying thyroid lesions into benign and malignant based on data captured using sonographic imaging. Accordingly, the aim of the present study was to determine whether radiomic imaging analysis can provide an accurate evaluation of thyroid nodules undergoing diagnostic US evaluation.

## 2. Materials and Methods

A systematic review was performed in accordance with the Preferred Reporting Items for Systematic Reviews and Meta-Analyses (PRISMA) guidelines [16] and in accordance with the Cochrane Handbook for Systematic Reviews of Diagnostic Test Accuracy [17]. Local institutional ethical approval was not required.

### 2.1. Population, Intervention, Comparison, Outcomes (PICO)

Population: Patients who have undergone preoperative US and definitive thyroid nodule diagnosis as benign or malignant.

Intervention: Radiomic analyses applied to preoperative US used to inform whether thyroid nodules are benign or malignant.

Comparison: The discriminative ability of radiomics compared to confirmation of benign and malignant nodules. Nodules were determined as benign by either cytological or histological means, while malignancy was confirmed by histological analysis only.

Outcomes: Primary outcomes included the evaluation of the clinical utility of preoperative US imaging to stratify thyroid nodules as either benign or malignant. Generated pooled sensitivity, specificity, and receiver operating characteristic (ROC) curve analyses will be representative of our primary outcomes. Secondary outcomes include comparing the ability of different radiomic methods to differentiate such nodules and to compare radiologists and radiomics in correctly discriminating benign versus malignant thyroid nodules.

### 2.2. Search Strategy

An electronic search of the PubMed Medline, EMBASE, and Scopus databases was performed on 16 January 2021 for relevant studies. This search was performed for the following headings: (Thyroid Cancer) and (Radiomics) linked using the boolean operator “AND”. Included studies were limited to those published in the English language and were not restricted based on the year of publication. All duplicate studies were manually removed before titles were screened, and studies deemed appropriate had their abstracts reviewed. Studies remaining had their full texts reviewed for eligibility.

### 2.3. Inclusion and Exclusion Criteria

Studies meeting the following inclusion criteria were included: (1) Studies with thyroid nodules confirmed as benign or malignant following US imaging; (2) imaging of tumors had to have been performed pre-diagnosis; (3) either stated study numbers of true positive, true negative, false positive, false negative, sensitivity, specificity, or accuracy data in relation radiomic tests or the ability to calculate these figures based on study data. In some cases, sensitivity and specificity were calculated from ROC curve analyses. Studies comparing the diagnostic ability of radiologists with radiomics were also included. Studies meeting any of the following exclusion criteria were excluded from this study: (1) studies not providing radiomic validation or “test” data, (2) studies outlining the diagnostic ability of radiomics differentiating benign and malignant lesions in other cancers (e.g., breast carcinoma, skin cancers, etc.), (3) studies with no full English text, (4) review articles, (5) studies including less than five patients in their series or case reports, and (6) editorial articles.

### 2.4. Data Extraction and Quality Assessment

This literature search was performed by two independent reviewers (E.F.C. and S.O.) using the aforementioned search strategy. Where discrepancies in opinion occurred between the reviewers, a third reviewer was asked to arbitrate (M.G.D.). As described, duplicate studies were removed. Both reviewers reviewed all retrieved manuscripts to ensure all inclusion criteria were met before extracting the following data: (1) first author name, (2) year of publication, (3) study design, (4) country, (5) level of evidence, (6) study title, (7) number of patients, (8) number of benign and malignant nodules confirmed though cytologic or histopathologic analysis, (9) sensitivity, specificity, and area under curve (AUC) scores from the ROC curve analyses obtained from radiomic “test” data and (10) sensitivity, specificity, and AUC scores from the ROC analyses from radiologists within studies where available. Sensitivity and specificity were directly extracted from tables and study text. When not provided as discrete data in tables or the text, specific estimates of sensitivity and specificity were calculated from ROC curves with the most accurate and appropriate sensitivity prioritized. Where studies tested the diagnostic ability of multiple radiomic methods (i.e., CNN, ML, etc.), only data for the best performing radiomic method within that study was extracted. Similarly, where studies detailed data on multiple radiologists’ ability to discriminate benign versus malignant nodules, data from the best performing radiologist from that particular study was included. Appraisal of the quality of each study was performed using the radiomics quality score (RQS), as outlined previously by Lambin et al. [18].

### 2.5. Statistical Analysis

Statistical analysis was performed according to the Cochrane guidelines. Pooled sensitivity and specificity and summary ROC analysis were calculated for included studies to demonstrate to convey the diagnostic test performance of radiomics in differentiating malignant thyroid nodules from benign thyroid nodules. We then performed a comparison between studies using CNNs (incorporating both CNNs and other deep learning methods) versus those using either ML or Radiomic AI analyses (together termed non-CNNs). For comparing radiologist and radiomic diagnostic test accuracy, sensitivity and specificity data were expressed as dichotomous data and reported as odds ratios (ORs) with 95% confidence intervals (CIs) following estimation using the Mantel–Haenszel method using random effects. The symmetry of funnel plots was used to assess publication bias. Statistical heterogeneity was determined using I2 statistics. Statistical significance was determined to be *p* < 0.05. Statistical analysis was performed using Review Manager (RevMan), Version 5.4 (Nordic Cochrane Centre, Copenhagen, Denmark).

## 3. Results

### 3.1. Literature Search

**Figure 1:**
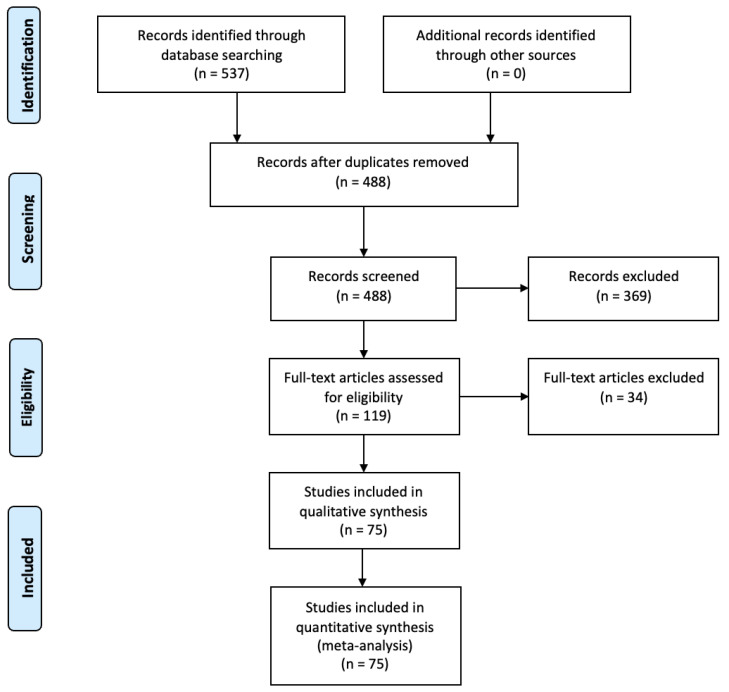
PRISMA flow diagram detailing the systematic search process.

The initial search of PUBMED, SCOPUS, and EMBASE resulted in a total of 537 studies identified. Following the removal of duplicates, 488 studies remained. These studies were then screened by title and abstract for relevance, after which 119 studies remained—all had their full text analyzed for eligibility. Finally, 75 studies remained for inclusion in the analysis as depicted by Figure 1 [19,20,21,22,23,24,25,26,27,28,29,30,31,32,33,34,35,36,37,38,39,40,41,42,43,44,45,46,47,48,49,50,51,52,53,54,55,56,57,58,59,60,61,62,63,64,65,66,67,68,69,70,71,72,73,74,75,76,77,78,79,80,81,82,83,84,85,86,87,88,89,90,91,92,93].

### 3.2. Study Characteristics

Overall, 75 studies arising from 15 different countries met inclusion and exclusion criteria (19–93), 8 studies were prospective in nature (25, 31, 38, 55, 57, 81, 83, 84) while the remaining 67 studies were retrospective. Of the included studies, 46 used convolutional neural networking (CNN) to analyse thyroid nodule US images (19, 25, 26, 29, 30, 33, 36–38, 40–45, 47, 48, 50–55, 57, 59, 63–65, 67–71, 73, 74, 77, 79, 81–84, 89–93), 29 studies used non CNN methods (20–24, 27, 28, 31, 32, 34, 35, 39, 46, 49, 56, 58, 60–62, 66, 72, 75, 76, 78, 80, 85–88) (Table 1).

### 3.3. Clinicopathological Characteristics

Overall, there were 28,373 patients with 46,175 thyroid nodules included from the 75 studies. Males accounted for 24.6% of patients, while 75.4% of patients were female. There were 51 studies reporting mean patient age; within these studies, mean patient age was 48.3 years (range: 42.2–69.0 years) (Table 1).

Overall, 22,814 (49.4%) nodules were benign while 23,361 (50.6%) of nodules were malignant. Within included studies, 35 reported mean nodule size; mean nodule size in these studies was 19.7 mm (range 8.3–31.7 mm). We found 34 studies provided a breakdown of malignant nodules by subtype. Papillary thyroid carcinoma (PTC) was the most prevalent subtype of malignant thyroid nodule within these studies, representing 94.7% of malignant thyroid nodules (Table 2).

### 3.4. Diagnostic Ability of Radiomics

**Figure 2:**
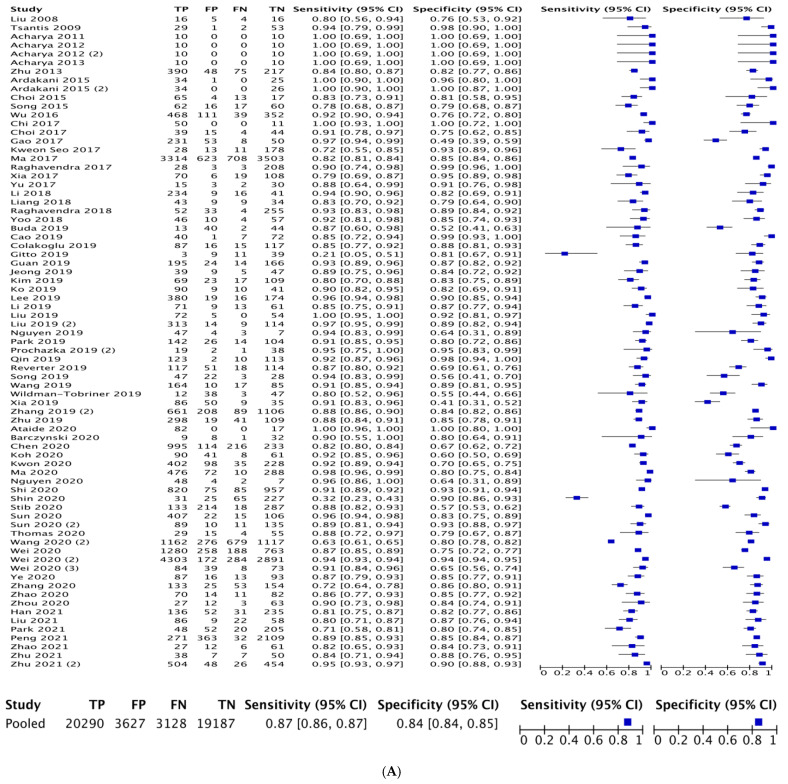

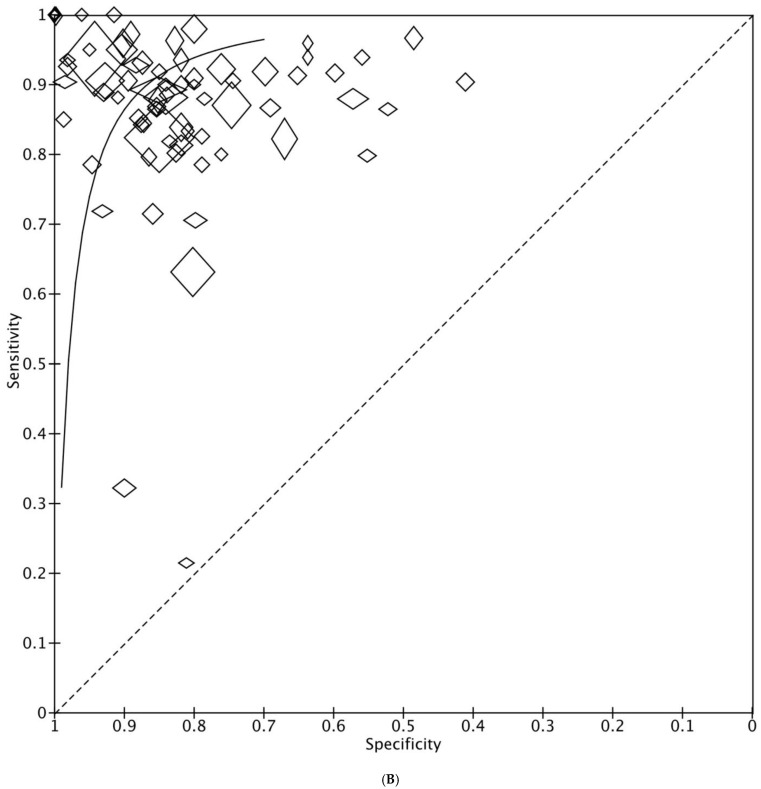
(**A**) Overall sensitivity and specificity of radiomics. (**B**) Receiver operating characteristic (ROC) curve of malignant versus benign thyroid nodules based on radiomic analyses.

The mean AUC calculated from independent ROC curve analyses within included studies was 0.88 (range: 0.61–1.00). Individual study sensitivity and specificity for determining malignant versus benign thyroid nodules is demonstrated in Figure 2A. Pooled sensitivity for radiomics in distinguishing thyroid nodules was 0.87 (95% CI: 0.86–0.87). Pooled specificity for radiomics in distinguishing thyroid nodules was 0.84 (95% CI: 0.84–0.85). A combined ROC curve for radiomics of thyroid nodules by ultrasound sonography is demonstrated in Figure 2B.

### 3.5. Comparison of CNN versus Non-CNN Radiomics

**Figure 3:**
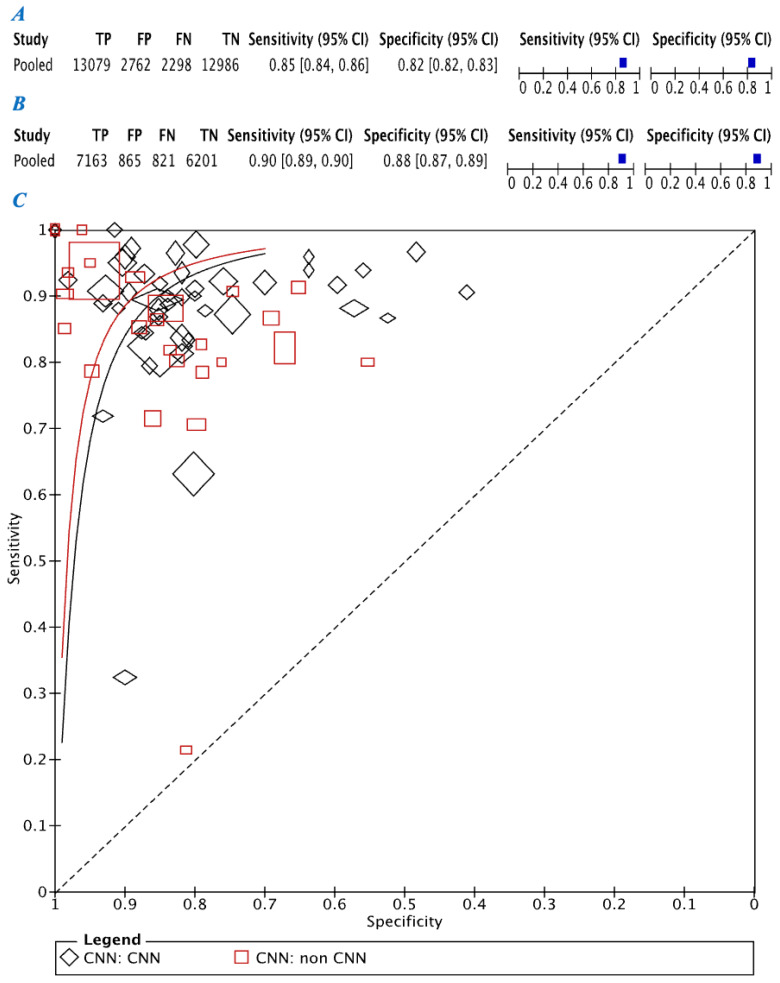
(**A**) Pooled sensitivity and specificity of convolutional neural network (CNN) analyses and (**B**) represents pooled sensitivity and specificity of non-CNN analyses. (**C**) Depicts the receiver operating characteristic (ROC) curve for CNN analyses (black) versus non-CNN analyses (red).

For studies using CNN pooled sensitivity was 0.85 (95% CI: 0.84–0.86) and pooled specificity was 0.82 (95% CI: 0.82–0.83). Pooled sensitivity 0.90 (95% CI: 0.89–0.90) and specificity 0.88 (95% CI: 0.87–0.89) was significantly higher in studies using non-CNN radiomics (*p* < 0.05) (Figure 3A,B). ROC curve comparison between CNN and non-CNN methods is outlined in Figure 3C.

### 3.6. Comparison of Radiomic Analysis of Thyroid Nodule US versus Radiologists Analysis of Thyroid Nodule US

**Figure 4:**
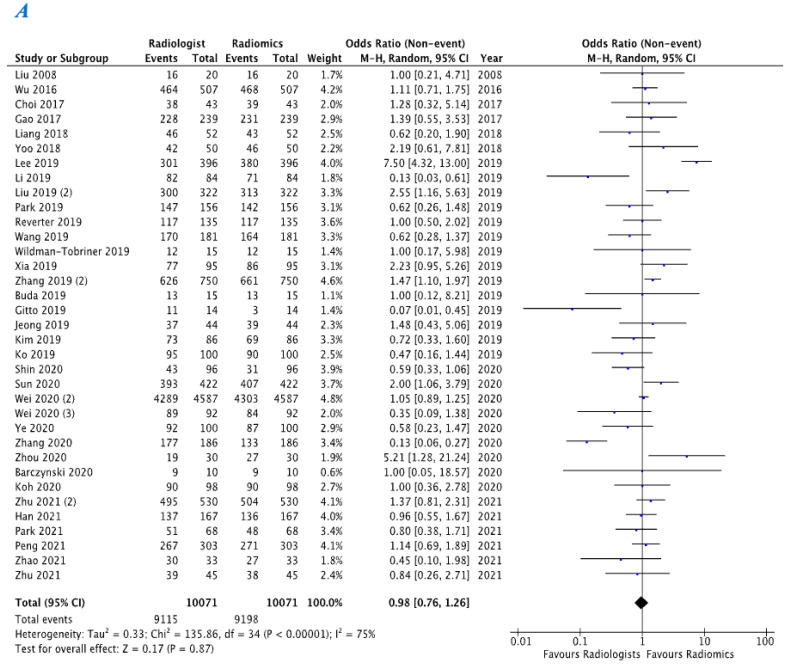

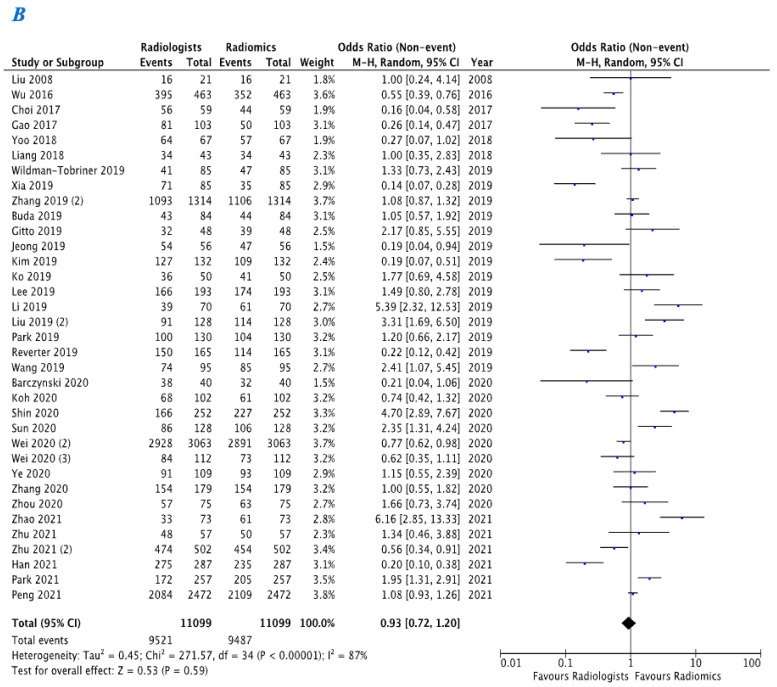
(**A**) Represents sensitivity comparison between radiologists and radiomics. (**B**) Represents specificity comparison between radiologists and radiomics.

Within the studies included in the meta-analysis, 35 studies provided a comparison between radiologists and radiomics in differentiating malignant versus benign thyroid nodules using thyroid US. Radiomics demonstrated similar sensitivity for detection of malignancy within a given thyroid nodule (OR 0.98, 95% CI 0.76–1.26) when compared with radiologists (Figure 4A). Radiomics also demonstrated similar specificity (OR 0.93, 95% CI 0.72–1.20) when compared with radiologists for this purpose (Figure 4B).

## 4. Discussion

To the best of our knowledge, the current systematic review and meta-analysis is the first to evaluate the diagnostic test accuracy of radiomic imaging analysis in differentiating malignant from benign thyroid nodules using US. Due to the increasing prevalence of thyroid nodules now detected within the general population and the rising incidence of thyroid malignancy (which has tripled since 1975), accurate risk stratification is paramount to the enhancement of clinical outcomes [3]. The most important finding in this analysis of over 28,000 patients possessing over 46,000 thyroid nodules is the data supporting the utility of radiomic analysis in correctly stratifying undetermined thyroid nodules correctly into benign and malignant lesions (sensitivity: 0.87, specificity: 0.84). This is promising as we look to enhance diagnostics in this field of oncology, all the while promoting minimally invasive techniques in order to reduce morbidity and mortality for prospective patients. These results come at the timely promotion of precision oncology as a rapidly evolving field, which manipulates individual patient, cancer, or disease process characteristics in order to develop a personalized diagnosis, prognosis, and treatment strategies [12]. Data from this analysis support radiomic imaging analysis using US as a means of quantification of malignancy in thyroid nodules, without exposing patients to the risks associated with invasive FNAC sampling or surgical specimen assessment. For some patients, the use of radiomics could possibly circumvent the need for FNAC and surgical resection, providing a potentially more cost and time-efficient assessment of thyroid nodules than what is currently practiced [20,94].

Results of this analysis indicate that radiomics is a novel avenue worth exploring in the differentiation of benign and malignant thyroid lesions. CNN provided a pooled sensitivity of 85% and specificity of 82% compared to a pooled sensitivity of 90% and pooled specificity of 88% in non-CNN. CNN is designed as an automated means to adaptively learn spatial hierarchies of features through backpropagation by using multiple building blocks: convolution layers, pooling layers, and fully connected layers of data processing [95]. CNN has powerful pattern recognition capabilities due to the fact that they can approximate any continuous function, given an appropriate network structure [96]. In neural networking, high variance gives networks the ability to learn complex patterns, although it also runs the risk of overfitting since models will learn peculiarities, or noise, from a data set [96]. The noise phenomenon incorporates features into the model which are not generalizable outside of the training set [95]. This makes the model appear to perform well in training but fail to perform in a true clinical environment. Such overfitting in the setting of CNN has been noted in studies evaluating papillary thyroid nodules on US [44]. The margin between benign thyroidal tissue and malignant tissue may be unclear or blurry on US imaging, with significant overlap between cancerous and normal or benign regions. Thus, it is then challenging for the CNN model to perform accurate textural feature extraction of the malignant tissue, possibly contributing to poor model performance [44]. Ideally, a CNN should have a large training set to mitigate the risk of overfitting, but this is not always feasible due to cost, time, and other factors limiting available data [95]. Non-CNN incorporates a number of methods such as support vector machines (SVM), random forest (RF), k-nearest neighbor (k-nn), and Bayesian classifiers [97]. Each method has its own strengths and weakness. For example, SVM classifiers are based on decision planes that define decision boundaries. SVM is often used for the principle of structural risk minimization, which allows robust analysis of test data without the need for a large training set through margin maximization [98]. Another popular ML method is RF, which consists of a large network of individual decision trees that allows for ensemble learning, providing the benefit of human-readable data and the ability to adjust the classifiers’ decision trees where appropriate [97]. Ultimately, the randomness of this model makes it robust, generalizable, and less prone to overfitting, although large numbers of decision trees make this approach more time-consuming. Within our analysis, small-data and overfitting within individual studies may have contributed to the overall worse performance of CNN versus non-CNN. Based on the results of this meta-analysis non-CNN radiomics should be the preferred methods for evaluating the risk of malignancy in an undetermined thyroid nodule using US.

For detecting malignancy within a given thyroid nodule radiomic methods had similar sensitivity (0.98, 95% CI 0.76–1.26) and specificity (0.93, 95% CI 0.72–1.20) when compared with radiologists. However, acknowledgment for the strengths maintained by radiologists compared to radiomics: At present, radiomic models are dependent on high-quality image acquisition and segmentation by radiologists. Without good imaging data to analyze, the radiomic model is unable to correctly stratify nodules [99]. Radiologists also maintain the innate ability to incorporate the global context of patients and the ability to maintain subjective associations based on experience, which current radiomic models are unable to perform. Radiomics can face issues with model fitting, poor input data, and subsequent suboptimal performance [100]. However, human assessment of medical imaging and, in particular, US suffers from significant inter-observer variability [101,102]. Radiomics, on the other hand, provides the benefit of an objective, quick, and reproducible analysis with the ability to analyze features of the nodule that are both visible to the radiologist and textural features occult to human perception [13,14]. Studies have attempted to blend the strengths of both radiologists and radiomic models to form computer-assisted diagnosis (CAD) tools. While CAD was not evaluated within the confines of the current meta-analysis, CAD has shown to be of benefit in the evaluation of thyroid nodules within the literature [39].

The present analysis is subject to a number of limitations. Primarily, radiomics involves a broad spectrum of analysis methods, ranging from the radiomic AI methods to deep-learning techniques; we have included all of these under the umbrella term “radiomics” despite variance in their reproducibility of data [103]. Secondly, the authors wish to highlight the inter-user variability of US due to this imaging acquisition being operator dependent. Radiomic analyses are dependent on high-quality images of thyroid nodules being obtained and nodules being correctly selected by ultra-sonographers. Thirdly, when extracting data, we selected the highest performing radiomic method within any given study. This may have led to over-estimation of overall sensitivity and specificity for radiomic evaluation of thyroid nodules on US as a whole. To combat this potential bias when comparing radiomics to radiologists, we selected data for the highest performing radiologist. Finally, prospective validation evaluating the utility of AI in the field of radiological diagnostics typically necessitates buying from large, international corporations in order to finance developing the evidence base in this field.

## 5. Conclusions

In conclusion, this meta-analysis of current evidence demonstrates an almost 90% reliability of radiomic imaging analyses to US in detecting malignancy within undetermined thyroid nodules. At present, radiomic analyses demonstrate equal diagnostic sensitivity and specificity of identifying malignant lesions when compared to radiologists. Within the field of radiomics, at present, non-CNN methods may be considered the preferred radiomic means of classifying thyroid nodules. Based on this meta-analysis, AI offers promising results as an avenue to be explored as we look to enhance the diagnostic accuracy and risk stratification of thyroid nodules in the era of personalized medical and oncological patient care. We advocate for rigorous experimentation in this field, given the potential for this technology to bolster diagnostic workflows, enhance clinical outcomes, and minimize patient morbidity; all while mitigating associated healthcare costs.

## Figures and Tables

**Table 1:** Study characteristics and demographics.

Author	Year	Study Type (LOE)	Radiomics	Country	US Device Brand	N Patients	Male	Female	Mean Age
Zhou	2020	RC (III)	CNN	China	Esaote/Phillips	105	25	80	47.9
Nguyen	2019	RC (III)	CNN	Korea	NS	61	NS	NS	NS
Wei	2020	RC (III)	CNN	China	NS	2489	614	1875	45.3
Park	2019	PC (II)	CNN	Korea	Samsung	265	52	213	47.1
Thomas	2020	RC (III)	CNN	USA	4 brands	103	NS	NS	NS
Wei (2)	2020	RC (III)	Non-CNN	China	5 brands	NS	NS	NS	47
Liu	2019	RC (III)	CNN	China	Vinno	131	54	77	46.7
Stib	2020	RC (III)	CNN	USA	Siemens/GE/Phillips	571	234	337	52.9
Ye	2020	RC (III)	CNN	China	5 brands	166	46	100	44.6
Ma	2020	RC (III)	CNN	China	NS	211	34	177	NS
Koh	2020	RC (III)	CNN	Korea	11 brands	200	49	151	49.6
Kwon	2020	RC (III)	CNN	Korea	Phillips/Hitachi	762	NS	NS	NS
Kim	2019	RC (III)	Non-CNN	Korea	Samsung	106	29	77	48
Zhao	2020	RC (III)	Non-CNN	China	Phillips/Hitachi	174	44	130	45
Qin	2019	RC (III)	CNN	China	NS	233	NS	NS	NS
Zhu	2021	RC (III)	CNN	China	4 brands	102	0	102	54.8
Liu (2)	2019	RC (III)	CNN	China	GE	376	NS	NS	NS
Xia	2019	PC (II)	CNN	China	Samsung	171	32	139	47.2
Zhao	2021	RC (III)	Non-CNN	China	SuperSonic	102	25	77	50.6
Lee	2019	RC (III)	CNN	Korea	Phillips/Hitachi	519	93	426	47.5
Ataide	2020	RC (III)	Non-CNN	Germany	NS	99	NS	NS	NS
Chen	2020	RC (III)	Non-CNN	China	GE/Hitachi	1480	302	1178	45.6
Zhu (2)	2021	RC (III)	CNN	China	Phillips/GE/Toshiba	261	64	197	52
Shi	2020	RC (III)	CNN	China	Esaote/Hitachi/Toshiba	NS	NS	NS	NS
Barczyński	2020	PC (II)	CNN	Poland	Samsung	50	9	41	47.5
Zhang	2020	RC (III)	Non-CNN	Korea	Siemens	303	59	244	46.4
Wei (3)	2020	RC (III)	Non-CNN	China	Samsung	181	35	146	46
Colakoglu	2019	RC (III)	Non-CNN	Turkey	GE	198	48	150	44.5
Park	2021	RC (III)	Non-CNN	Korea	Phillips	325	61	264	50.1
Nguyen	2020	RC (III)	CNN	Korea	NS	61	NS	NS	NS
Sun	2020	RC (III)	CNN	China	GE	338	134	416	43.8
Peng	2021	PC (II)	CNN	China	13 brands	2775	726	2049	42.2
Liu	2021	RC (III)	CNN	China	Siemens	163	48	115	44.3
Han	2021	RC (III)	CNN	Korea	Samsung	372	NS	NS	NS
Shin	2020	RC (III)	CNN	Korea	Samsung	340	79	261	47.2
Wang	2019	RC (III)	CNN	China	GE/Phillips	276	53	223	46.3
Zhu	2019	RC (III)	CNN	China	4 brands	467	97	370	45.3
Zhang (2)	2019	RC (III)	Non-CNN	China	Hitachi	2032	695	1337	42.3
Ko	2019	RC (III)	CNN	Korea	Phillips/Hitachi	150	23	127	49.7
Song	2019	RC (III)	CNN	Korea	Toshiba	100	NS	NS	NS
Li	2019	RC (III)	CNN	China	Phillips/Toshiba/GE	154	34	120	51
Wildman-Tobriner	2019	RC (III)	Non-CNN	UK	Siemens/GE/Phillips	94	21	73	52.6
Yu	2017	PC (II)	CNN	China	Phillips/Siemens	50	9	41	48.4
Buda	2019	RC (III)	CNN	USA	Siemens/GE/Phillips	91	NS	NS	52.3
Raghavendra	2018	RC (III)	Non-CNN	India	4 brands	344	NS	NS	44.1
Li	2018	RC (III)	CNN	China	NS	300	53	247	NS
Ma	2017	RC (III)	CNN	china	7 brands	4782	NS	NS	52
Raghavendra	2017	RC (III)	Non-CNN	India	GE	242	63	179	44.1
Zhu	2013	RC (III)	CNN	China	Siemens	618	161	528	47.7
Choi	2017	PC (II)	Non-CNN	Korea	Samsung	89	18	71	43.5
Gao	2017	RC (III)	CNN	China	Phillips/GE	342	70	272	44.8
Choi	2015	RC (III)	CNN	Korea	Phillips	85	24	61	52
Chi	2017	RC (III)	CNN	Canada	Toshiba	61	NS	NS	NS
Jeong	2019	PC (II)	CNN	Korea	Samsung	76	NS	NS	NS
Acharya	2012	RC (III)	Non-CNN	Singapore	NS	20	10	10	NS
Liang	2018	RC (III)	Non-CNN	China	Phillips	95	20	75	43.2
Prochazka (2)	2019	RC (III)	Non-CNN	Czechia	Phillips/GE	60	11	49	55.7
Song	2015	RC (III)	Non-CNN	China	GE	147	32	115	NS
Ardakani	2015	RC (III)	Non-CNN	Iran	Medison	60	NS	NS	NS
Guan	2019	RC (III)	CNN	China	NS	399	NS	NS	NS
Xia	2017	RC (III)	Non-CNN	China	Siemens	187	36	151	50.8
Yoo	2018	PC (II)	CNN	Korea	Samsung	50	10	40	43.2
Tsantis	2009	RC (III)	Non-CNN	Greece	Phillips	85	NS	NS	NS
Liu	2008	RC (III)	Non-CNN	USA	NS	37	NS	NS	NS
Acharya	2013	RC (III)	Non-CNN	Italy	Esaote	20	10	10	52.8
Acharya (2)	2012	RC (III)	Non-CNN	Italy	Esaote	20	10	10	52.8
Acharya	2011	RC (III)	Non-CNN	Italy	Esaote	20	10	10	52.8
Kweon Seo	2017	RC (III)	CNN	Korea	NS	230	51	179	48.7
Ardakani (2)	2015	RC (III)	CNN	Iran	Medison	60	NS	NS	NS
Wu	2016	RC (III)	CNN	China	Phillips	970	214	756	46.7
Cao	2019	RC (III)	Non-CNN	China	NS	120	NS	NS	NS
Wang (2)	2020	RC (III)	CNN	China	NS	1040	NS	NS	NS
Sun (2)	2020	RC (III)	CNN	China	NS	245	NS	NS	NS
Reverter	2019	RC (III)	Non-CNN	Spain	GE	300	45	255	55.5
Gitto	2019	RC (III)	Non-CNN	Italy	Samsung	62	12	50	60

NS: not specified, LOE: level of evidence, RC: retrospective cohort, PC: prospective cohort, CNN: convolutional neural network, non-CNN: analysis performed using a method other than a convolutional neural network, GE: General Electric.

**Table 2:** Study characteristics and demographics.

Author	Year	N Nodules	Mean Nodule Size (mm)	N Benign Nodules	N Malignant Nodules	Papillary Ca	Follicular Ca	Medullary Ca	Other Thyroid Ca
Zhou	2020	105	NS	75	30	NS	NS	NS	NS
Nguyen	2019	61	NS	11	50	NS	NS	NS	NS
Wei	2020	2489	NS	1021	1468	1442	11	15	0
Park	2019	286	16.2	130	156	149	6	1	0
Thomas	2020	103	NS	70	33	24	3	2	4
Wei (2)	2020	7560	NS	3063	4587	NS	NS	NS	NS
Liu	2019	131	16.1	59	72	72	0	0	0
Stib	2020	651	NS	500	151	NS	NS	NS	NS
Ye	2020	209	NS	109	100	NS	NS	NS	NS
Ma	2020	846	NS	360	486	NS	NS	NS	NS
Koh	2020	200	22.4	102	98	97	0	0	1
Kwon	2020	762	NS	325	437	437	0	0	0
Kim	2019	218	12	132	86	86	0	0	0
Zhao	2020	177	21.8	96	81	81	0	0	0
Qin	2019	248	NS	115	133	NS	NS	NS	NS
Zhu	2021	NS	NS	57	45	NS	NS	NS	NS
Liu (2)	2019	450	NS	128	322	NS	NS	NS	NS
Xia	2019	180	10.3	85	95	91	4	0	0
Zhao	2021	106	17.3	73	33	NS	NS	NS	NS
Lee	2019	589	12.9	193	396	395	1	0	0
Ataide	2020	99	NS	17	82	NS	NS	NS	NS
Chen	2020	1558	NS	347	1211	NS	NS	NS	NS
Zhu (2)	2021	1032	NS	502	530	NS	NS	NS	NS
Shi	2020	1937	NS	1032	905	NS	NS	NS	NS
Barczyński	2020	NS	30.5	40	10	10	0	0	0
Zhang	2020	365	18.3	179	186	168	11	7	0
Wei (3)	2020	204	15	112	92	90	1	0	1
Colakoglu	2019	235	NS	133	102	102	0	0	0
Park	2021	325	21	257	68	NS	NS	NS	NS
Nguyen	2020	NS	NS	11	50	NS	NS	NS	NS
Sun	2020	550	14	128	422	NS	NS	NS	NS
Peng	2021	2775	NS	2472	303	299	4	0	0
Liu	2021	175	11.9	67	108	103	5	0	0
Han	2021	454	17.8	287	167	161	4	2	0
Shin	2020	348	31	252	96	0	96	0	0
Wang	2019	NS	18.5	95	181	NS	NS	NS	NS
Zhu	2019	467	8.3	128	339	NS	NS	NS	NS
Zhang (2)	2019	2064	NS	1314	750	NS	NS	NS	NS
Ko	2019	150	12.9	50	100	NS	NS	NS	NS
Song	2019	100	NS	50	50	NS	NS	NS	NS
Li	2019	154	NS	70	84	NS	NS	NS	NS
Wildman-Tobriner	2019	100	27.1	85	15	NS	NS	NS	NS
Yu	2017	50	NS	33	17	16	0	1	0
Buda	2019	99	27	84	15	NS	NS	NS	NS
Raghavendra	2018	344	NS	288	56	NS	NS	NS	NS
Li	2018	NS	NS	50	250	250	0	0	0
Ma	2017	8148	25	4126	4022	NS	NS	NS	NS
Raghavendra	2017	242	NS	211	31	NS	NS	NS	NS
Zhu	2013	689	13.3	265	465	NS	NS	NS	NS
Choi	2017	102	12	59	43	43	0	0	0
Gao	2017	342	12.1	103	239	NS	NS	NS	NS
Choi	2015	99	NS	21	78	77	1	0	9
Chi	2017	NS	NS	11	50	NS	NS	NS	NS
Jeong	2019	100	17	56	44	43	1	0	0
Acharya	2012	20	NS	10	10	7	1	0	2
Liang	2018	95	16	43	52	51	1	0	0
Prochazka (2)	2019	60	NS	40	20	NS	NS	NS	NS
Song	2015	155	NS	76	79	NS	NS	NS	NS
Ardakani	2015	60	NS	26	34	NS	NS	NS	NS
Guan	2019	399	NS	190	209	209	0	0	0
Xia	2017	203	24.8	114	89	NS	NS	NS	NS
Yoo	2018	117	15	67	50	50	0	0	0
Tsantis	2009	85	NS	54	31	NS	NS	NS	NS
Liu	2008	41	NS	21	20	18	0	0	2
Acharya	2013	20	31.7	10	10	7	1	0	2
Acharya (2)	2012	20	31.7	10	10	7	1	0	2
Acharya	2011	20	31.7	10	10	7	1	0	2
Kweon Seo	2017	230	29.4	191	39	0	39	0	0
Ardakani (2)	2015	60	NS	26	34	NS	NS	NS	NS
Wu	2016	970	NS	463	507	487	12	4	4
Cao	2019	120	NS	73	47	NS	NS	NS	NS
Wang (2)	2020	3120	NS	1393	1841	NS	NS	NS	NS
Sun (2)	2020	245	NS	145	100	NS	NS	NS	NS
Reverter	2019	300	29.8	165	135	112	15	3	5
Gitto	2019	62	18	48	14	NS	NS	NS	NS

NS: not specified, Ca: cancer.

## Data Availability

Data used within this manuscript is available online via Pubmed, EMBASE, and Scopus.
